# Supplementary material for: Self-care knowledge, attitude and associated factors among outpatients with diabetes mellitus in Arsi Zone, Southeast Ethiopia
Source: PLOS Glob Public Health. 2021 Dec 28;1(12):e0000097. doi: 10.1371/journal.pgph.0000097 (PMC10021671; doi:10.1371/journal.pgph.0000097)
Supplement: Attachment — Submitted filename: Rebuttal letter to the reviewers & editors.docx [file pgph.0000097.s001.docx]

**Rebuttal Letter for the reviewers**

**Reviewer #1**: I want to express my heartfelt thanks to the author for their nice endeavor on a very burning issue at lower middle income country context. But i found few confusing parts all over the study. My comments regarding the study is below

1. Authors included both type 1 and type 2 DM. About 290 patients from OPD settings. Patient selection criteria is confusing and not that clear. Line no 157 is so much confusing.

**Reply: Patient selection criteria and the inclusion of both type 1 and type 2 DM**

If we are clear with the question regarding patient selection criteria, we accept that the comment is valuable and needs clarification, the table that was attached in the manuscript at line 108 is not clearly described and makes some confusion. We have tried to make it clear and described below. We didn’t consider of the proportional allocation of the type 1 and type 2 diabetes patients before data collection. Actually it was not the objective of the study. However, we categorized the type of the diabetes mellitus after data collection period of time with their corresponding size. Accordingly, 169(58.3%) type 1 and 121(41.7%) type 2 diabetes patients were involved in this study.

The inclusion criteria were being a patient with both type 1 & type 2 diabetes mellitus and age of 18 years old and above. The other criterion was that having an outpatient department follow-up for at least the last six months before data collection.

The monthly flow of the patients was determined from the health management and information system (HMIS) of each hospital (Asella Referral Hospital, Arsi Robe Hospital, Abomsa Hospital and Bekoji Hospital) found in Arsi zone. The total number of the patient flow from those four hospitals found in Arsi zone was 507, 371, 322, and 302 respectively. The number of study participants was proportionally allocated for each hospital to maintain the representativeness of the data (99, 71, 62, and 58 respectively). The total summation of these numbers gives the number of the sample size that was two hundred ninety (290). Every fifth patient was selected using the systematic random sampling technique for interview. Simple random sampling or lottery method was used to select the first patient to start the interview.

2. How much time is required for interviewing per patients in that OPD settings?

**Reply:** The interview took about 30 minutes. The time allocated for each respondent during interview was 30 minute and inserted in the manuscript.
3. At the study, majority of the participants was from rural area and 21.7% were not able to write and read. But analysis is showing that more than half of the participants had good self care knowledge. So, how the authors want to explain that situation as it is not normal. I did not find any proper judgment on discussion regarding this issue.

**Reply:** The finding on this study showed that majority of respondents had good knowledge towards DM self-care even though, most of the respondents were from rural and 21.7 % were unable to write and read. The authors clearly mentioned on the discussion part on line number 212-218.

This could be due to the reason that the study was a hospital based and the sample populations were DM patients on follow up at hospital/specifically at the diabetic clinic so that they were better exposed for health education and interpersonal experience sharing among patients themselves and from health care provider that in turn increase their level of understanding and knowledge.

4.How the authors classified the socio-economic background as poor, medium and rich? There is no explanation on this context. Have they used World Bank classification or anything other else scale?

**Reply:**The reference for the socioeconomic background classification used was and inserted in the manuscript. The classification was made based on world bank GDP of the household’s and classified extreme poverty (households spending below $1.90 per person per day) and those in the middle class (households spending $11-110 per day per person in 2011 purchasing power parity, or PPP) and those who are at the top of the distribution who are classified as “rich.”

Besides, similar literatures were used as a reference (Niguse H, *et al, 2019*)

5. Is there any explanation from the author’sside, why type 1 DM patients are more than 50% on their study as because it is not normal? We know about 90% of DM are type 2. So, it needs clarification.

**Reply: The question is quite right and scientific. Even though it is known that Type 1 diabetes affects children and adults, people can be diagnosed at any age (Juvenile Diabetes Research Foundation,** [**https://www.jdrf.org/t1d-resources/about/facts/**](https://www.jdrf.org/t1d-resources/about/facts/)**, 2021)**

**Similarly, in this study, the majority (63.2%) of the study participants were from the age group of 18 to 44 that are the young adults.**

**On the other hand, this study justifies that 200(66.9%) of them were using insulin that possibly indicates that most of the patients in the study had diagnosed with type 1 diabetes even though some patients with type 2 diabetes would also take insulin if oral anti diabetes agents fail to lower the glucose level in the blood.**

**On the other hand, we do have a plan to conduct further study on a research question “why type 1 diabetes mellitus became more prevalent than type 2 DM on this study area?”**
6. Age categorization and distribution on analysis showed some irrelevancy. Most of the patients were at that group where they should not be. So, how authors will evaluate this?

**Reply**: Right you are, it has a bit deviation from the standard age classification developed by World Health Organization (WHO). However, since this standard categorization starts its classification from the age group of 15-19, it fails to include our study’s’ first classification which was 18-24. Here in Ethiopia the adult category starts with the age of 18 years old.

On the other hand, if we follow the standard classification, the age categories in the study is not conducive for analysis and may not be inclusive.

7. Analysis showed 15.5% students had DM. What is their type of DM? It should be mentioned.

**Reply: About ninety five (95.6%) of the students were developed type 1 Diabetes mellitus.**
8. How have the authors evaluated the following situations of diabetic complications?

a. Have they diagnosed retinopathy? What was the retinal evaluation procedure?
b. Have they searched for diabetic neuropathy?

**Reply: All the diabetic complications were searched or collected from the patient chart due to resource constraints**.
9. Duration of Diagnosis of DM and age grouping of DM is confusing and contradictory.

**Reply: If we are clear with the question;** the duration of diagnosis of DM is defined as the years that the patient experienced after diagnosis of DM at the time of data collection and categorized in to three groups:

1. <5 years
2. 5-10 years
3. >10 years

10. It can be easily described 'why the patients had poor attitude?". So, normally we can expect who have good knowledge, may have good attitude. This explanation is missing from the study.

**Reply:** **Though the relationship between knowledge and attitude are discussed with different findings, additional justification is included in the discussion.**

**The present study revealed that most of the respondents had unfavorable attitude though most of them had good knowledge. This could be due to the fact that good knowledge does not necessarily guarantee that the patient will have good attitude. As different scientific researches show people who have better knowledge are more resistance and questionable rather than implementing what they know rather than those who know less. Knowledgeable members of the public are in general, less supportive of morally contentious areas than those who are less knowledgeable.**

**Reviewer #2:** Please change the title. Knowledge and attitude towards diabetes not self-care practices in method, it should be described more details. Other comments are included in the manuscript. Please check. Follow those instructions and suggestions.

**Reply: It is a good comment. However, we better state as it is since the aim of this study as a whole intends to describe or wants to investigate the level of knowledge and attitude of the patients towards the diabetes self care related to the disease rather than towards the disease itself only.**

**As you suggested us, we will describe or confirm the relationship of the variables or the ideas under the method in details to make it clearer.**

**Title:** Self care knowledge, attitude and its associated factors among out patients with diabetes mellitus in Arsi zone, South East Ethiopia

**Abstract:**

**Line number 39**: Conclusion: the explanation of the findings or results which was described in number is avoided and summarized like the following.

Majority of the study participants had good knowledge towards diabetes self-care practice while a significant number of the participants had not favorable attitude towards diabetes self care.

**Line number 79**: Materials and Methods

The patients included under the study were old diagnosed diabetes patients at least with six months old after diagnosis.

The references for the literatures from which the interview questions developed are the reference number 15 and 16.

**Line number 100: Study participants &selection method**

The comment is constructive that it is known that the old diagnosed patients would have the information about diabetes and its self-care. However, as a researcher, we have to know or confirm the level of the knowledge and attitude regarding the self-care activities related to Diabetes to make it scientific so that the scientific communities could use it for different purpose. Moreover, it could help different stakeholders to use the findings to take measures accordingly.

On the other hand, in the developing countries like in Ethiopia, it is difficult to give health education/information specific to the disease for all patients on follow up where limited resources, particularly the health care provider: patient ratio is not balanced even for the patients who came for follow up to the health institution.

**Operational definition and the data collection tool:** the Likert scale was not used, and the questionnaire is adapted and contextualized to the situations in our countries set up. It is a validated questionnaire from the beginning and a pretest was done after adaptation to our context. The following references like**” Measuring the attitudes of patients towards diabetes and its treatment, Diabetes Attitude Survey. Diabetes Research and training center. 1998”was used.**

**The cut-off point or reference to categorize the knowledge and attitude towards the self care was taken from previous studies done on similar topics that were used similar tool or questionnaire(**Niguse, H., Belay, G., Fisseha, G. *et al.* Self-care related knowledge, attitude, practice and associated factors among patients with diabetes in Ayder Comprehensive Specialized Hospital, North Ethiopia. *BMC Res Notes* **12,** 34 (2019). https://doi.org/10.1186/s13104-019-4072-z)

**Line no 160**: Wrong numbering of the table.

We have corrected it: It is corrected as Table 2

**Line number 209**: “This study tried to assess ~~self-care practice~~ knowledge and attitude among diabetic patients in Arsi zone and 64.9%, 26.8% of the respondents had good knowledge and good attitude towards self-care respectively”.

**The comment on the above statement is valuable that you suggested to state like attitude towards diabetes only not on the self-care. However, we better describe the objective or the intention of the study which is about the level /status of the knowledge and attitude of the patients towards the diabetes self care rather than towards the disease itself only.**

**Additional corrections from the authors**

1. Grammatical and editorial problems are corrected as much as possible; and included both under the manuscript with track changes and clear manuscript
2. Table 1. Is rejected
3. Cross tabulations is done for Table 3& 4 to know the exact distribution of the independent variables with related to the dependent variable.
