# Supplementary material for: Self-care knowledge, attitude and associated factors among outpatients with diabetes mellitus in Arsi Zone, Southeast Ethiopia
Source: PLOS Glob Public Health. 2021 Dec 28;1(12):e0000097. doi: 10.1371/journal.pgph.0000097 (PMC10021671; doi:10.1371/journal.pgph.0000097)
Supplement: Attachment — Submitted filename: Rebuttal letter reviewers.docx [file pgph.0000097.s002.docx]

**Rebuttal Letter for the reviewers**

**Both Reviewer #1** $ #2 commented that the manuscript has changed rigorously and all comments were approached. Finally, they recommended it for submission. However, reviewers and the editor recommended that to insert symbolic marks to the tables and to define them later under the table.

Under Table 1.

a. The variable “Body mass index” was not stated using it measurement and know we put it measurement as “ Kg /m^2 “^
b. The variable “Duration of diagnosis” is stated with its time measurement which is “in years”

Under Table 3 & Table 4.

a. The sterics (*) are defined under the tables.

- We have checked for the references & as to our best level of knowledge

**Note: Reviewer #2 attached some comments through attachment. But, the attachment was with the previous manuscript which was not edited.**
